# A rare partnership: patient community and industry collaboration to shape the impact of real-world evidence on the rare disease ecosystem

**DOI:** 10.1186/s13023-024-03262-2

**Published:** 2024-07-10

**Authors:** T. L. Klein, J. Bender, S. Bolton, T. Collin-Histed, A. Daher, L. De Baere, D. Dong, J. Hopkin, J. Johnson, T. Lai, M. Pavlou, T. Schaller, I. Žnidar

**Affiliations:** 1 National MPS Society, PO Box 14686, Durham, NC USA; 2 International MPS Network, Ottawa, Ontario, Canada; 3 Sanofi, Cambridge, MA USA; 4 International Niemann-Pick Disease Registry (INPDR), Newcastle, UK; 5 International Gaucher Alliance (IGA), London, UK; 6 Gaucher Registry for Development, Innovation & Analysis of Neuronopathic Disease (GARDIAN), London, UK; 7 Casa Hunter – Brazilian Association of Hunter Disease Patients and Other Rare Diseases, São Paulo, Brazil; 8 Fabry International Network (FIN), Antwerp, Belgium; 9 National Niemann-Pick Disease Foundation (NNPDF), Rochester, NY USA; 10 Fabry Support & Information Group (FSIG), Concordia, MO USA; 11 Hong Kong Mucopolysaccharidoses & Rare Genetic Diseases Mutual Aid Group (HKMPS), Kowloon, Hong Kong; 12 International Pompe Association (IPA), Baarn, The Netherlands; 13 Pompe Deutschland eV, Weingarten (Baden), Germany

**Keywords:** Fabry disease, Gaucher disease, Pompe disease, MPS I, Lysosomal storage diseases, Patient communities, Registries, Real-world data, Real-world evidence

## Abstract

People with rare lysosomal storage diseases face challenges in their care that arise from disease complexity and heterogeneity, compounded by many healthcare professionals being unfamiliar with these diseases. These challenges can result in long diagnostic journeys and inadequate care. Over 30 years ago, the Rare Disease Registries for Gaucher, Fabry, Mucopolysaccharidosis type I and Pompe diseases were established to address knowledge gaps in disease natural history, clinical manifestations of disease and treatment outcomes. Evidence generated from the real-world data collected in these registries supports multiple stakeholders, including patients, healthcare providers, drug developers, researchers and regulators. To maximise the impact of real-world evidence from these registries, engagement and collaboration with the patient communities is essential. To this end, the Rare Disease Registries Patient Council was established in 2019 as a partnership between the Rare Disease Registries and global and local patient advocacy groups to share perspectives on how registry data are used and disseminated. The Patient Council has resulted in a number of patient initiatives including patient representation at Rare Disease Registries advisory boards; development of plain language summaries of registry publications to increase availability of real-world evidence to patient communities; and implementation of digital innovations such as electronic patient-reported outcomes, and patient-facing registry reports and electronic consent (in development), all to enhance patient engagement. The Patient Council is building on the foundations of industry–patient advocacy group collaboration to fully integrate patient communities in decision-making and co-create solutions for the rare disease community.

**Graphical Abstract:**

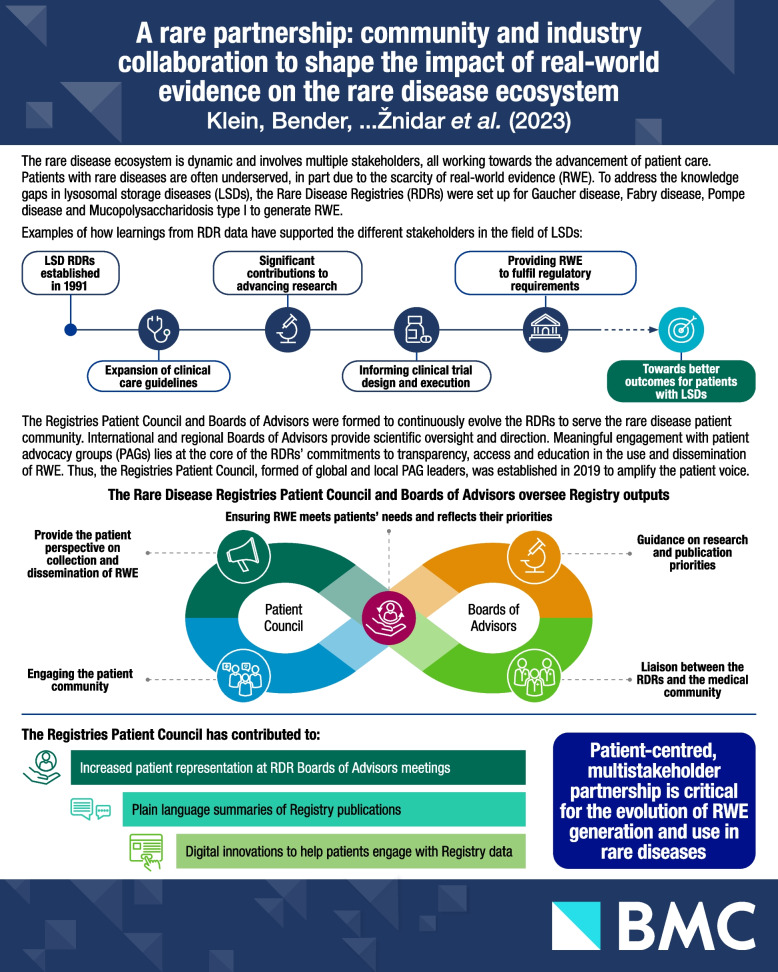

**Supplementary Information:**

The online version contains supplementary material available at 10.1186/s13023-024-03262-2.

## Background

There is no universally accepted definition of ‘rare disease’. In the European Union, a disease is defined as rare if it affects fewer than 1 in 2,000 people [1]. In the United States, the Orphan Drug Act defines a rare disease as one that affects fewer than 200,000 people in the population, while Japan uses 50,000 people as its threshold [2, 3]. Around 7,000 identified rare diseases affect approximately 350 million people worldwide [4]. Their poorly defined disease prevalence and the heterogeneity of rare diseases pose unique challenges across all aspects of the patient journey, patient care and drug development [4].

Lysosomal storage diseases (LSDs) are a group of rare, inherited metabolic disorders generally caused by deficiencies in lysosomal enzymes which result in defective substrate breakdown, leading to build-up in affected organs [5–7]. Substrate accumulation causes organ dysfunction and a broad spectrum of clinical manifestations. There are around 70 known LSDs. While they are individually rare, their collective incidence varies from approximately 1 in 5,000 to 1 in 7,000 births, dependent on population [6].

In addition to significant morbidity, people with LSDs often have long diagnostic odysseys, which may be compounded by inadequate disease understanding across healthcare providers [6]. Consequently, some patients have difficulty finding specialised care and/or have limited treatment options. Adding further complexity, LSDs have various genotypes and phenotypes, with a variable extent of disease severity. These factors have led to challenges in collecting robust data on disease natural history, management and treatment outcomes [8].

The Rare Disease Registries (RDRs) were established in 1991 with the International Collaborative Gaucher Group (ICGG) Registry, followed by the Fabry Registry in 2001, Mucopolysaccharidosis type I (MPS I) Registry in 2003 and Pompe Registry in 2004. We will describe the RDRs and the establishment of the RDR Patient Council in the context of successful partnerships of patient advocacy groups (PAGs) and industry, and outline the work undertaken by the RDR Patient Council to date.

### Overview of the Rare Disease Registries

The history, process and impact of the RDRs were reviewed in 2022 by Mistry et al. [8]. The RDRs were initiated to address the unmet need of collecting real-world data (RWD) to generate real-world evidence (RWE) on the natural history of LSDs and treatment outcomes [8]. These data are especially important in LSDs due to a limited knowledge of disease natural history, disease heterogeneity and small patient populations. Expert-endorsed management guidelines are often limited and specialised centres are usually geographically dispersed [9]. Moreover, standard clinical trial designs may not be feasible and modifications to statistical tests, endpoints, dosing and multi-arm designs may be necessary to achieve sufficient statistical power [10].

The RDRs represent the largest, global, observational databases for Gaucher disease, Fabry disease, MPS I and Pompe disease. They are the result of a collaborative partnership between members of the rare disease community, including healthcare professionals (HCPs), statisticians, patients, PAGs and Sanofi. These registries provide a mechanism for collecting RWD on disease signs and symptoms, clinical assessments, patient-reported outcomes (PROs) and treatment outcomes. All patients with a confirmed diagnosis are eligible to participate, regardless of their therapy status and choice of treatment. Patients may be enrolled at any time in the course of their disease through participating registry sites. Patients receive standard-of-care treatment as determined by their physicians, who then enter patients’ longitudinal data [8].

**Fig. 1:**
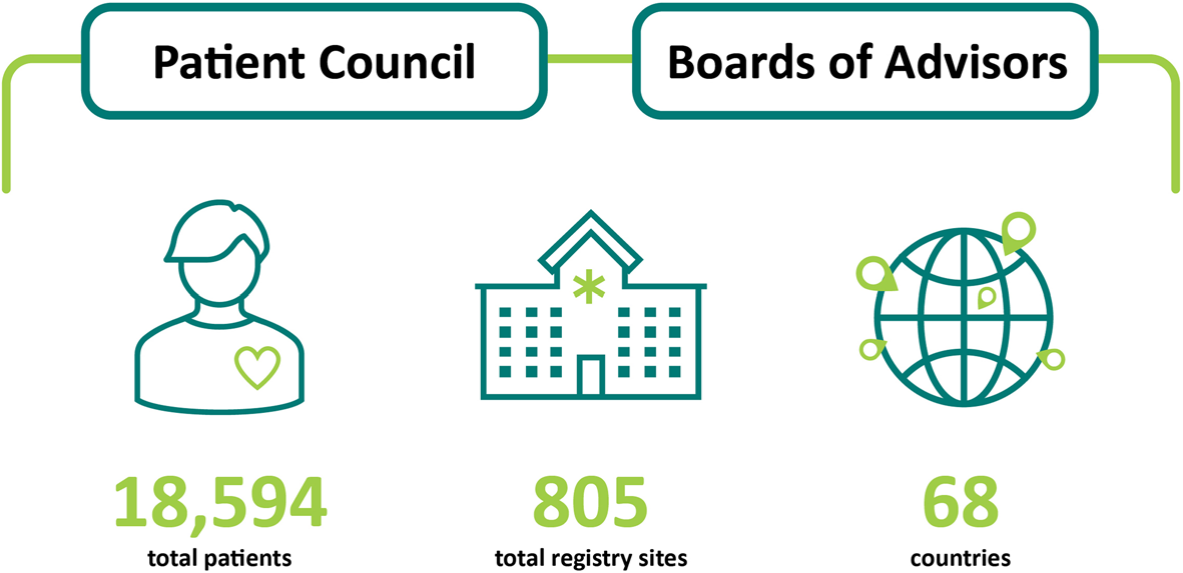
Rare Disease Registries in numbers [8]

Since the introduction of RDRs in 1991, more than 18,000 patients have enrolled at over 800 sites in 64 countries (see Fig. 1), resulting in an increased capacity to generate RWE which will only grow as enrolment continues. In turn, to date, more than 100 peer-reviewed articles have been published using RDR data. This wealth of data has increased the understanding of disease natural history, clinical characteristics, genotype–phenotype correlations, comorbidities and treatment outcomes for diseases such as Gaucher, Fabry, MPS I and Pompe [8, 11]. For example, data from the ICGG Gaucher Registry showed that Gaucher disease is a systemic disorder that is not limited to the macrophage system, where patients have an increased risk of malignancy and of developing Parkinson’s disease/Lewy body dementia [12], and delineated the impact of different variants in *GBA* and their relationships with subtypes of Gaucher disease [13]. Similarly, data from the Fabry Registry revealed that approximately 5% of patients with Fabry disease (FD) experienced major cardiovascular events if they did not start treatment or prior to initiating enzyme replacement therapy [14], leading to the requirement for patients with FD to be monitored for cardiovascular risk factors. The Fabry Registry also transformed our understanding of disease manifestations in female patients: previously, female patients with heterozygous mutations in *GLA* were thought to be asymptomatic carriers [15, 16]. Registry data showed that they are at high risk for major organ involvement and a decreased quality of life due to random X-chromosome inactivation [16].

Moreover, learnings based on Registry data have resulted in improvements in diagnosis, facilitating earlier initiation of treatment, and in the expansion of patient-monitoring guidelines [12]. For example, data from the MPS I Registry showed that time to treatment has shortened significantly since the development of new therapies, leading to an improvement in patient outcomes [17]; the data were also used to develop comprehensive clinical management guidelines, aiding in increasing awareness of MPS I, early identification of symptoms and long-term monitoring [12].

Furthermore, outside the clinical and research communities, RDRs have been a rich source of data for regulatory authorities, payors, health technology assessment bodies and policymakers [8, 11]. Registry data have been used in regulatory decision-making and post-marketing authorisation assessments by the European Medicines Agency and the US Food and Drug Administration [18–20]. Thus, regulators and other decision-makers can leverage registry data as a part of the evidence used in governing decisions for approvals, drug access and reimbursements [19]. Using RWE to support post-marketing authorisation applications helps expedite patient treatment availability, ultimately improving patient outcomes [18–20]. Additionally, registries provide a platform for collaboration and data exchange between international regulators in evaluating evidence for treatments of rare diseases, including clinical trial design, risk-management plans and early-access mechanisms [18, 20].

### Patient advocacy groups in rare disease

**Fig. 2:**
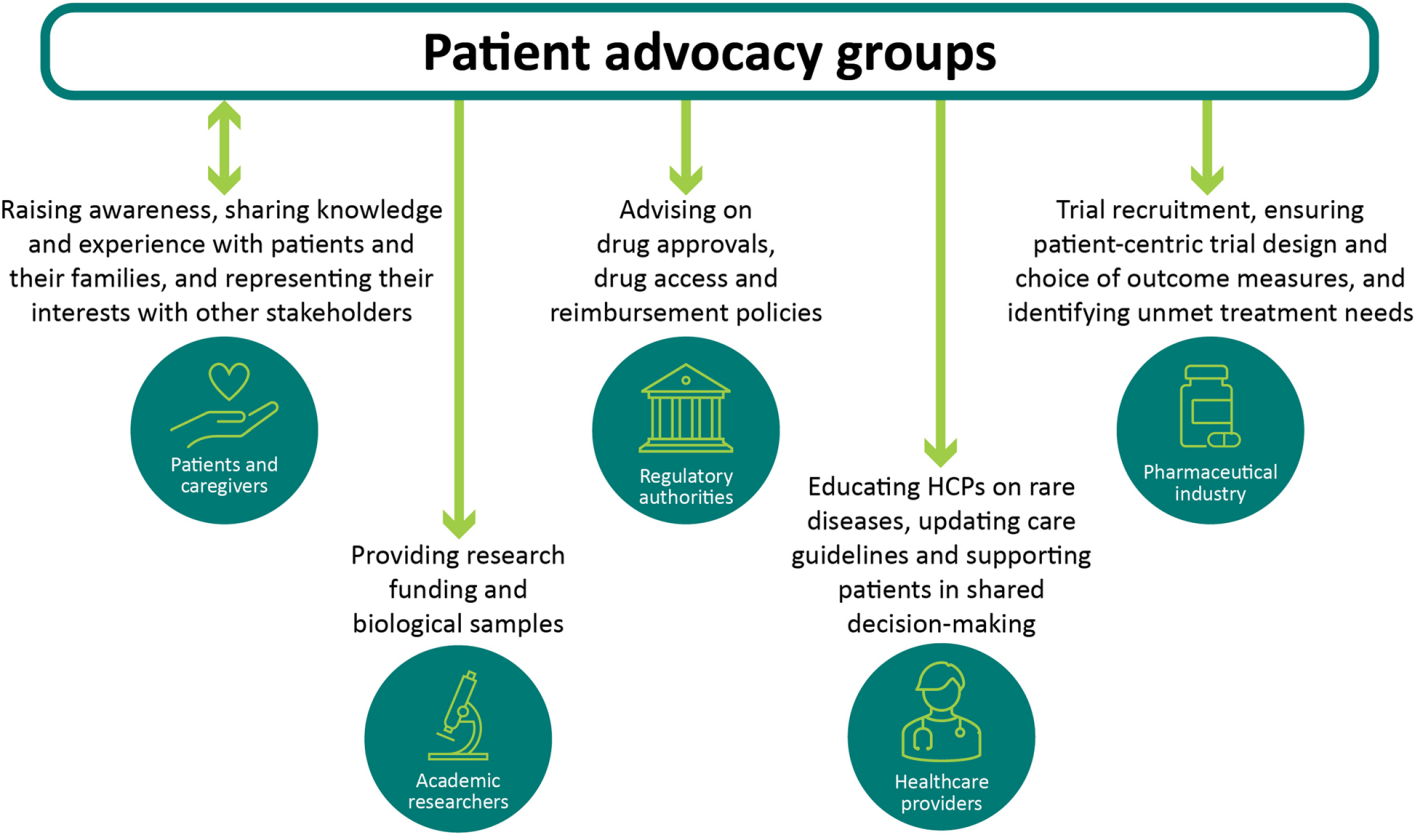
Interactions patient advocacy groups have with the various other stakeholders in the rare disease ecosystem HCP, healthcare professional

PAGs provide patients and their families with information, education and support, connect them with available resources, defend and represent their interests [21]. Their roles have evolved significantly as they have become key partners in the rare disease ecosystem, working closely with healthcare providers, pharmaceutical companies, regulatory authorities and academics to best serve the patient community [4]. The roles of PAGs in the field of rare disease are summarised in Fig. 2. In a survey of 159 international rare disease PAGs, it was reported that the most common annual budget for running their activities, serving 1,000–10,000 members, was between USD 100,000 and USD 200,000. Most funding was obtained through charitable donations, fundraising events and corporate sponsorship [21].

PAGs are critical in providing disease-specific education to HCPs and other clinical team members who may not regularly encounter patients with rare diseases [22]. PAGs also empower patients to take a more active role in shared decision-making [4] (see Fig. 2).

In drug development, PAGs have significantly contributed to patient recruitment, clinical trial protocol design, identification of endpoints and PROs [23–27]. For instance, after the inception of the International Pompe Association (IPA) in 1999, members could engage with and directly fund preliminary research into enzyme replacement therapies (ERTs). The IPA played a key role in advocating for commercialisation and approval of an ERT and supported in updating management guidelines [28].

In relation to the collection of RWD, PAGs are vital collaborators to ensure the RWE generated is meaningful to patients and addresses their needs effectively. Insights from PAGs and their communities can be used to drive patient-centric practices in data collection, thus enhancing the quality of RWE [29, 30]. Given that PAGs are a primary source of information for patients, these organisations are ideally placed to lead patient education efforts and improve the standards of RWD collection and increase patient understanding of the value of RWE [31]. The patient experience can provide pertinent information that may be missed by researchers or clinicians, for example on activities of daily living, concomitant medication use or the effect of socioeconomic factors [30]. PAGs have also driven the adoption of patient-facing summaries and outputs of Registry data, a key effort to aid patients in understanding why their data are being collected [31, 32].

PAGs promote patient data ownership and address issues of transparency and data access. PAGs have also created independent, patient-led registries, including two organisations that are members of the RDR Patient Council: the Gaucher Registry for Development, Innovation and Analysis of Neuronopathic Disease (GARDIAN), which collects clinical and patient-relevant outcomes, and the International Niemann-Pick Disease Registry (INPDR), which contributes to consensus clinical management guidelines and assessments of clinical disease characteristics [11, 12, 33, 34].

Moreover, the ARthritis Partnership with Comparative Effectiveness Researchers Registry (AR-PoWER) and DuchenneConnect Registry also exemplify PAG–registry partnerships where patient input shapes the processes of informed consent, PRO selection and response to patient enquiries [29]. By its very nature, RWE can be an important tool in fostering a sense of community, as it captures longitudinal data from diverse patient populations, regardless of inclusion criteria used in clinical trials. Patients are able to become part of a community and see how RWD collection benefits them individually and collectively [30].

## The Rare Disease Registries Patient Council

### Overview of the Rare Disease Registries and Patient Council partnership

The RDR Patient Council was established in 2019 to build on successful collaborations between PAGs and the various stakeholders in the rare disease community. It plays a key role in the continued evolution and expansion of the RDRs. The RDRs are guided by the Boards of Advisors (BoAs), consisting of physicians (experts in the respective disease), patient representatives and the Patient Council (Fig. 1). The last of these is comprised of global and local PAG leaders who consult on patient needs and engage with the wider patient community. The physicians at the regional and international BoAs use their clinical and scientific expertise to direct the Registries’ research and publication priorities.

**Fig. 3:**
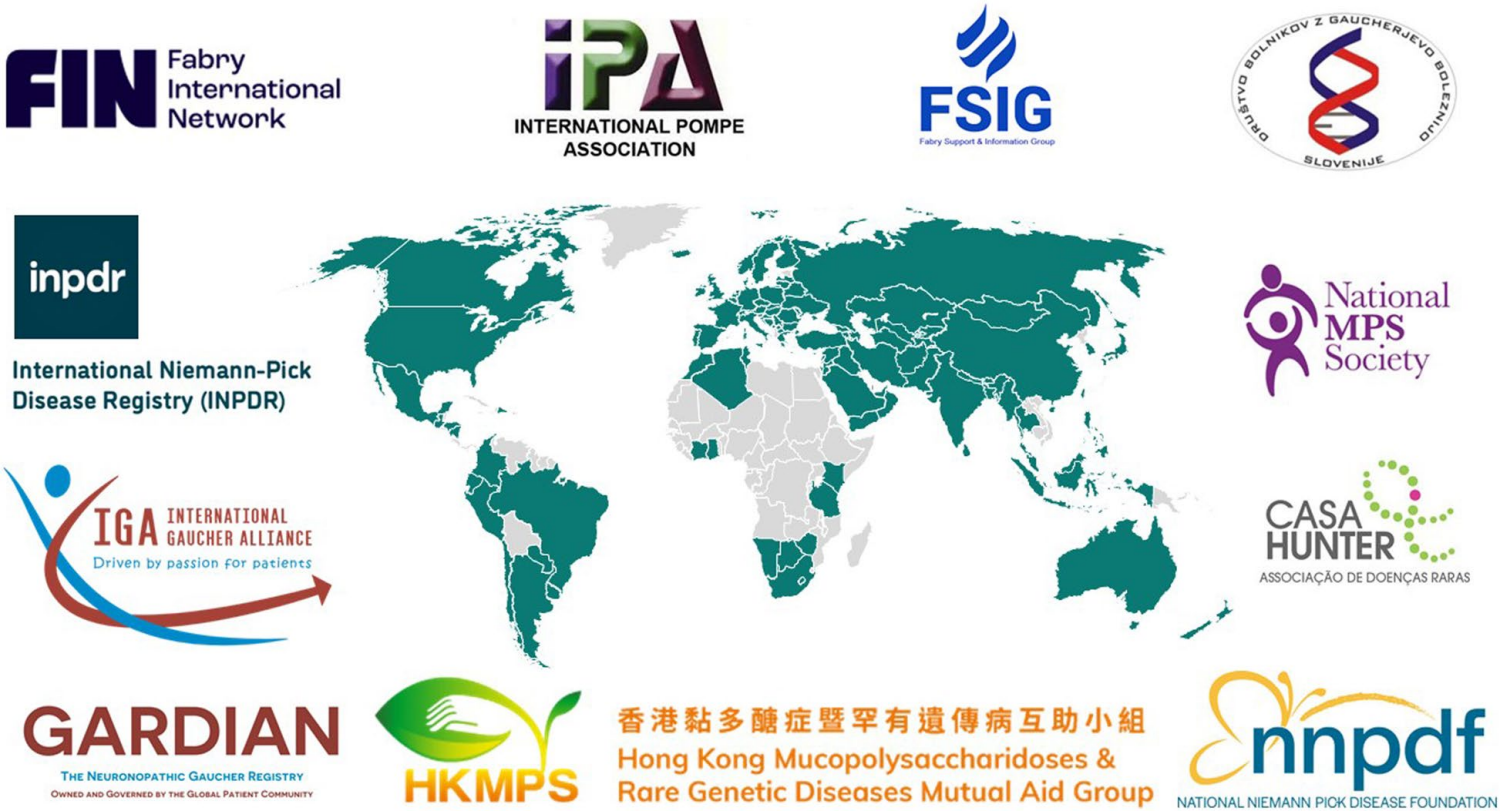
Global reach of the patient advocacy groups represented in the Rare Disease Registries Patient Council

The RDR Patient Council was established to increase patient engagement and collaborate with global and local PAGs. It was founded in 2019 to partner the RDRs with the patient community in the use and dissemination of RWE. The PAGs represented in the RDR Patient Council have a wide-reaching global impact, supporting patients in more than 100 countries (Fig. 3).

The RDR Patient Council is also an avenue for co-creating solutions with the patient community, and it has identified RWE as an important topic for continued dissemination of information to the wider patient community as well as to other stakeholders. Education on the relevance of RWE in rare diseases is invaluable for HCPs and patients alike. Through engagement in the RDR Patient Council, PAGs can provide greater understanding of the RDRs for their organisations, including why their data are collected and how those data will be used. Therefore, by involving patients, the Registries can achieve the shared goals of transparency, access, education and maximising the use of RWD to produce evidence on rare diseases. Multistakeholder engagement and collaboration are critical across registries, whether the model is industry-sponsored or patient-led.

### Rare Disease Registries Patient Council aims and initiatives

The RDR Patient Council set out to achieve the following: 1) increasing the visibility and utility of the RDRs to stakeholders, 2) fostering the identity of the RDR Patient Council and 3) amplifying patient engagement with the RDRs. These aims share the common purpose of involving patients in leveraging RWD to increase data literacy and generate purposeful RWE that can be used to inform decisions to improve patient outcomes.

The RDR Patient Council and the ongoing partnership established with the member organisations brought about several initiatives to shape the way evidence is generated and disseminated for and with the patient community. These include PAG representation at BoA meetings, plain language summaries (PLSs) of registry publications, digital innovations for increased patient participation, and the development of educational materials. Furthermore, the RDR Patient Council serves as a platform for cross-organisational dialogue between patient- and industry-led Registries. It provides a forum to share best practices and learnings, as well as methods of optimum collaboration.

To enhance the visibility of the RDRs and patient engagement with Registry activities, the RDR Patient Council advocated greater patient representation at BoA meetings. Since 2021, PAG leaders have been present at the Fabry, Gaucher, MPS I and Pompe Registry international BoA meetings. Since 2023, PAG leaders have also been present at regional BoA meetings.

#### Patient-facing plain language summaries

The RDR Patient Council championed the drive to tailor communication channels used to disseminate Registry publications and to widen their reach to a larger cross-section of the rare disease community. The result has been the development and publication of peer-reviewed, open-access PLSs of Registry publications available in English, Chinese, French, German, Italian and Spanish [35, 36]. These PLSs are peer-reviewed by a patient who does not have the disease being presented, to ensure they are easily understood by the patient community. PLSs are summarised in Table 1.

**Table 1:** Plain language summaries produced using Registries data as effective patient education tools

Registry	PLS title	Content and impact	Number of downloads	Reference
Fabry Registry	Plain language summary of a study looking at heart muscle thickness and kidney function in women with Fabry disease who received agalsidase beta treatment	• Engaging infographic summary of Registry data results elucidating the impact of Fabry disease on female patients • Females with heterozygous mutations in *GLA* (the causative gene for Fabry) were assumed to be asymptomatic carriers • Findings showed that females with Fabry disease should be monitored for cardiovascular events, kidney function and quality of life	2,453	[37]
Gaucher Registry	Plain language summary of the International Collaborative Gaucher Group Gaucher Risk Assessment for Fracture score in people living with Gaucher Disease Type 1	• Used tables and diagrams to present data on bone fracture risks from the Gaucher Registry • Outlined effective tools for measuring fracture risk and the effect of treatment • Identified patient groups at higher risk of developing fractures	1,006	[38]
Pompe Registry	Plain language summary: How the Pompe Registry is helping to identify and explain gene changes in Pompe disease	• Presented the geographical distribution of *GAA* variants and their relationship to disease severity based on Pompe Registry data, using maps and illustrations	1,014	[39]
MPS I Registry	Plain language summary of a study looking at the age at diagnosis and time to start of treatment in individuals with mucopolysaccharidosis type I (MPS I)	• Visually demonstrated long-term Registry data on the age of diagnosis and time to treatment initiation • Highlighted the need for improved disease awareness and earlier diagnosis • Supported the inclusion of MPS I in newborn screening programmes	1,097	[40]

Download metrics accurate as of November 2023

*MPSI* mucopolysaccharidosis type I, *PLS* plain language summary

PLSs present the publications in language suitable for the patient community and wider public, free from technical terminology or complex statistics. PLSs based on Registry publications also use visual aids to explain disease pathophysiology, symptoms, inheritance patterns and treatment options. They are an effective patient education tool and support informed patient participation in clinical decision-making and treatment personalisation. PLSs from each of the four Registries have been well received, with more than 5,500 downloads (Table 1) [37–40]. Topics for future PLSs were also suggested by the Patient Council, as well as methods to increase patient reach and accessibility, such as improving online navigation to the PLSs and exploring the possibility of including them on Registry websites. The Patient Council made recommendations on tailoring communication channels to patients in different geographies to improve awareness of the published PLSs via open access on the journals’ websites.

#### Patient accounts and electronic patient-reported outcomes

Other initiatives established and endorsed to increase patient participation are digital innovations that enable patients to engage with the RDRs and enter their data directly and easily. These include patient accounts to enter electronic patient-reported outcomes (ePROs) and quality-of-life and disease-burden data; an example of this is the inclusion of the 36-Item Short Form Health Survey (SF-36). These ePROs can be expanded further by adding sections on comorbidities and other drugs taken. The Patient Council advised that moving on from paper-based entries would increase patient participation. Additional enhancements include interactive Global Registry Reports, patient clinical summaries and electronic consent (eConsent) forms.

These digital innovations can support the integration of patient-centred health outcomes and disease-burden data with clinical data entered by physicians. This information can then be used to generate patient-facing reports and enhance the patient experience. Patient accounts with access to ePROs and Registry reports are currently in jurisdictions where allowable under local regulations. To maximise the impact of these data, the RDR Patient Council emphasised the future possible need to include the caregiver perspective for paediatric patients and patients with severe disease, in addition to developing educational materials. The Patient Council also provided recommendations on simplifying this process by integrating data collection with physician visits and reducing survey length to increase participation.

#### Interactive Global Registry Reports and patient clinical summaries

The RDR Patient Council was involved in the review of patient-facing registry outputs, such as Global Registry Reports and Patient Clinical Summaries, which are available via patient accounts to enrolled registry participants. Global Registry Reports present filterable aggregate data including enrolment numbers and disease-specific summaries of data, such as age at onset and age at first treatment. The Patient Council advised on optimal approaches to raising patient awareness of the platform and additional data for inclusion in future updates, such as comorbidities, all disease-associated symptoms, genetic variants and geographical locations of patients. Members also requested updates to RDR infographics highlighting currently participating sites in addition to the available infographics that summarise all historical enrolling sites, plus adding visuals illustrating the RDRs’ growth over time. Moreover, the RDR Patient Council affirmed the need for accessibility, (e.g., via one-click access) and translation into multiple languages as well as adding a filter-by-country option, for patients to see the impact of sharing their data. The RDR Patient Council also advocated for transparency of reporting, noting that this would empower patients to feel part of a global community while functioning as an educational resource for patients and their caregivers.

To promote the functionality of the Registries for clinicians and patient communities, updates to the graphical and tabular representation of individual patient-level data available to the patient and their Registry physician via their Registry accounts were endorsed and validated by the RDR Patient Council. The International Gaucher Alliance (IGA), a member organisation, offered to set up dedicated boards of patients to review patient accounts and the resultant reports, ensuring their suitability. There are current plans to expand this initiative beyond IGA, to the rest of the participating PAGs. The RDR Patient Council recognised that clinical, laboratory and treatment data can be used as tools that patients can share with their HCPs on a regular basis, furthering patient participation in shared decision-making. Furthermore, the RDR Patient Council affirmed that innovative, complex methodologies can be applied to the RWD collected in the RDRs to generate valuable RWE to augment the body of evidence in rare disease where every study is important. Thus, the RDR Patient Council noted that patients should be integrated into the review of Registry study designs, and that the Patient Council could serve as a place where the patient perspective on how studies are performed, including statistical methodologies used to produce RWE, will ensure relevance to and understanding by the patient community.

#### Electronic consent

The RDRs are developing eConsent forms, a digital platform to collect informed consent from patients where permitted. These will facilitate enrolment in the RDRs, streamline administrative processing and reduce burden on patients and caregivers where local regulations allow for the tool’s implementation.

The RDR Patient Council supported the development of eConsent forms and highlighted methods to employ them effectively and communicate their utility to patient communities. Technical support, e-learning modules and training videos were encouraged by the RDR Patient Council to ensure patients fully understood the process. Privacy and data protection remain a priority for patients, and the RDR Patient Council was able to provide reassurance by highlighting the various security measures taken to safeguard patients’ data.

The RDR Patient Council members recognise that patient engagement with Registries is key to maximising the potential of the Registries and therefore encourage their communities to do so. Creation of Registry materials, such as brochures and infographics, provides resources that describe the Registries at the consumer level, clarifying how patients’ data are used, increasing transparency and addressing concerns about the potential lack of detailed feedback they receive as participants. Ease of access to Registry reports and clinical summaries will also help engage the community and demonstrate the benefit of the Registries. Insights from the Council that will be carried forward include elucidating the differences between clinical trials and Registries and organising workshops or webinars for members.

## Conclusion

Rare diseases are characterised by a lack of RWE, which may result in suboptimal care for patients. It is therefore imperative that patients are involved in shaping how this evidence is generated and used, as key stakeholders and the end users of therapies. The RDRs Patient Council is a successful example of a partnership that engages the patient community to help raise awareness and understanding of the importance of registries and RWD/RWE and the crucial role that patients play.

As disease understanding grows, there is an increased appreciation of and demand for RWE to support access to existing treatments and development of new treatments. Registries, in general, and the RDRs in particular, provide critical, long-term data needed to inform all key stakeholders in the rare disease ecosystem. Effective multistakeholder collaboration, including close partnerships with patients and their representatives, is crucial to maximising the value of RWE.

### Supplementary Information

Supplementary Material 1.

## Data Availability

Not applicable.

## Abbreviations

AR-PoWER: ARthritis Partnership with Comparative Effectiveness Researchers Registry
BoA: Board of Advisors
eConsent: electronic consent
ePRO: electronic patient-reported outcome
ERT: enzyme replacement therapy
GARDIAN: Gaucher Registry for Development, Innovation and Analysis of Neuronopathic Disease
HCP: healthcare professional
ICGG: International Collaborative Gaucher Group
IGA: International Gaucher Alliance
INPDR: International Niemann-Pick Disease Registry
IPA: International Pompe Association
LSD: lysosomal storage disease
MPS I: mucopolysaccharidosis type I
PAG: patient advocacy group
PLS: plain language summary
PRO: patient-reported outcome
RDR: Rare Disease Registry
RWD: real-world data
RWE: real-world evidence
